# Garlic consumption in relation to colorectal cancer risk and to alterations of blood bacterial DNA

**DOI:** 10.1007/s00394-023-03110-2

**Published:** 2023-04-24

**Authors:** Michela Carola Speciani, Giorgio Gargari, Roberto Penagini, Massimiliano Mutignani, Monica Ferraroni, Arianna Natale, Michail Katsoulis, Marcello Cintolo, Pierfrancesco Leone, Aldo Airoldi, Maurizio Vecchi, Rossella Bonzi, Clorinda Ciafardini, Barbara Oreggia, Pietro Carnevali, Simone Guglielmetti, Patrizia Riso, Carlo La Vecchia, Marta Rossi

**Affiliations:** 1 Branch of Medical Statistics, Biometry, and Epidemiology “G. A. Maccacaro”, Department of Clinical Sciences and Community Health, Università Degli Studi Di Milano, Via Celoria 22, 20133 Milan, Italy; 2 Department of Food, Environmental and Nutritional Sciences (DeFENS), University of Milan, Milan, Italy; 3 Gastroenterology and Endoscopy Unit, Fondazione Istituto di Ricovero e Cura a Carattere Scientifico (IRCCS), Ca’ Granda Ospedale Maggiore Policlinico, 20122 Milan, Italy; 4 Department of Pathophysiology and Transplantation, University of Milan, Milan, Italy; 5 Digestive and Interventional Endoscopy Unit, ASST Grande Ospedale Metropolitano Niguarda, Milan, Italy; 6 MRC Unit for Lifelong Health and Ageing, Institute of Cardiovascular Science, UCL, London, UK; 7 General Surgery Unit, Foundation IRCCS Ca’ Granda Ospedale Maggiore Policlinico, Milan, Italy; 8 Hepatology and Gastroenterology Unit, ASST Grande Ospedale Metropolitano Niguarda, Milan, Italy; 9 Division of Minimally-Invasive Surgical Oncology, Niguarda Cancer Center, ASST Grande Ospedale Metropolitano Niguarda, Milan, Italy

**Keywords:** Blood microbiome, Garlic consumption, Colorectal cancer, Intestinal adenoma, 16S rRNA gene profiling, Insulin resistance

## Abstract

**Purpose:**

Garlic consumption has been inversely associated to intestinal adenoma (IA) and colorectal cancer (CRC) risk, although evidence is not consistent. Gut microbiota has been implied in CRC pathogenesis and is also influenced by garlic consumption. We analyzed whether dietary garlic influence CRC risk and bacterial DNA in blood.

**Methods:**

We conducted a case–control study in Italy involving 100 incident CRC cases, 100 IA and 100 healthy controls matched by center, sex and age. We used a validated food frequency questionnaire to assess dietary habits and garlic consumption. Blood bacterial DNA profile was estimated using qPCR and16S rRNA gene profiling. We derived odds ratios (ORs) and the corresponding 95% confidence intervals (CIs) of IA and CRC according to garlic consumption from multiple conditional logistic regression. We used Mann–Whitney and chi-square tests to evaluate taxa differences in abundance and prevalence.

**Results:**

The OR of CRC for medium/high versus low/null garlic consumption was 0.27 (95% CI = 0.11–0.66). Differences in garlic consumption were found for selected blood bacterial taxa. Medium/high garlic consumption was associated to an increase of Corynebacteriales order, Nocardiaceae family and *Rhodococcus* genus, and to a decrease of Family XI and *Finegoldia* genus.

**Conclusions:**

The study adds data on the protective effect of dietary garlic on CRC risk. Moreover, it supports evidence of a translocation of bacterial material to bloodstream and corroborates the hypothesis of a diet-microbiota axis as a mechanism behind the role of garlic in CRC prevention.

**Supplementary Information:**

The online version contains supplementary material available at 10.1007/s00394-023-03110-2.

## Introduction

Colorectal cancer (CRC) is the second leading cause of cancer death worldwide [1], with mortality rates still rising for young adults in various countries including the US and the UK [2]. More than half of CRCs in the US has been attributed to modifiable risk factors such as physical inactivity, tobacco smoking and diet [3]. Overweight and obesity are other crucial risk factors for CRC and a high consumption of plant based foods have been related to CRC risk reduction [4, 5]. In particular, garlic (*Allium sativum*) has been inversely associated to CRC risk in case–control studies, although results for cohort studies are inconsistent [6, 7].

Garlic is a source of non-digestible carbohydrates [8] and contains polyphenols and organosulfur compounds [9], which were related with a lower risk of CRC [10, 11]. Given its antioxidant, anti-inflammatory [12, 13] and antibacterial properties [14], garlic has been suggested to influence gut microbiota and the health of the intestinal mucosa [15–17], and can impact on epithelial permeability.

Increasing evidence points out the presence of bacterial DNA in the blood of healthy individuals, commonly referred to as “blood microbiome”, overcoming the traditional idea of blood as a sterile environment other than in case of pathogenic events [18]. Indeed, the presence of genetic bacterial material in blood has been associated with bacterial translocation from the intestinal tract to systemic circulation through the epithelial barrier [19], especially in conditions affecting the intestinal mucosa such as inflammatory bowel disease and CRC [20–22].

The aim of our study is to assess the relation between garlic consumption and CRC risk in an Italian setting, and to evaluate whether garlic consumption is associated to a blood bacterial DNA profile, using qPCR and16S rRNA gene profiling.

## Methods

Data come from a case–control study conducted between 2017 and 2019 in two university hospitals in the metropolitan area of Milan, Italy [20].

Participants were recruited among eligible outpatients or inpatients scheduled for a colonoscopy, also from CRC screening program. Among exclusion criteria were: selected inflammatory diseases, immunodeficiency, liver/kidney/heart failure, reported previous cancer, recent hospitalization or colonoscopy and dietary modifications in the previous month. Intestinal adenoma (IA) and IA/CRC free subjects were excluded if any colonic endoscopic resection had been previously performed.

Two pathologists revised colonoscopy and histological examinations and determined CRC cases and their clinical characteristics, as well as IA and subjects free from IA/CRC (hereafter referred to as “healthy controls”).

The sample includes 300 subjects: 100 incident, histologically confirmed CRC, 100 IA patients and 100 healthy controls, frequency-matched with cases by study center, sex and age ± 5 years. CRC patients (62 men and 38 women) had a mean age of 67 (range 31–85), IA patients 66 (range 34–84) and controls 66 years (range 26–85).

Cases included 21 cancers in the right colon (International Classification of Diseases, 10th Edition, ICD-10, C18.0, C18.2, C18.3), 12 in the transverse colon, splenic flexure, and descending colon (ICD-10, C18.4, C18.5, C18.6), 17 in the sigmoid colon (ICD-10, C18.7), and 50 in the rectum, including the rectosigmoid junction (ICD-10, C20, C19.9).

Less than 2% refused to participate in the study, and about 50 subjects were excluded during the enrollment procedures. All participants signed the written consent of the study. The protocol was approved by the ethical committees of the involved hospitals: ASST Grande Ospedale Metropolitano Niguarda (No. 477–112,016) and Fondazione IRCCS Ca' Granda Ospedale Maggiore Policlinico (No. 742–2017).

### Interview

A structured questionnaire was administered by trained interviewers, including information on socio-demographics, education, anthropometric measures, and lifestyle habits, such as smoking and physical activity. Patients’ usual diet before colonoscopy was collected through a food frequency questionnaire (FFQ) tested for reproducibility and validity [23], including 75 items on foods or Italian recipes and 5 on alcoholic beverages. Another section included both quantitative and qualitative questions referring to general dietary practices, including a closed question asking for the common garlic consumption. We scored low/null consumption of garlic for subjects reporting garlic use for flavoring but not for eating, and medium/high consumption for subjects reporting garlic use for flavoring and occasionally or frequently eating.

### Blood collection

Blood samples were collected before the colonoscopy in an aliquot of 7 mL in EDTA and immediately stored at – 80 °C. One microvial of 1 mL from each subject was sent to Vaiomer SAS, Labège, France, for the microbiota analysis. The samples from all subjects were analyzed in the same experiment and operators were blind to the group assignment.

### DNA extraction, quantitative polymerase chain reaction (qPCR) experiments and sequencing of 16S rRNA gene amplicons

Vaiomer SAS used an optimized blood-specific technique to quantify bacterial DNA and sequence reaction [19, 20, 24]. DNA was extracted from 0.25 mL of whole blood and collected in a final 50 μL extraction volume. Real-time PCR amplification was performed using panbacterial primers EUBF 5′-TCCTACGGGAGGCAGCAGT-3′ and EUBR 5′-GGACTACCAGGGTATCTAATCCTGTT-3′ [25], which target the V3-V4 hypervariable regions of the bacterial 16S rRNA gene with 100% specificity (i.e., no eukaryotic, mitochondrial, or Archaea DNA) and high sensitivity (16S rRNA of more than 95% of bacteria in Ribosomal Database Project database were amplified). The abundance of the 16S rRNA gene in blood samples was measured by qPCR in triplicate and normalized using a plasmid-based standard range. The results were expressed in number of copies of 16S rRNA gene per µl of blood. DNA from whole blood was then used to apply MiSeq Illumina technology for 16S rRNA gene taxonomic profiling. Four samples (2 IA and 2 control subjects) did not reach the threshold of 5000 reads and were excluded from taxonomic profiling analysis [20].

Vaiomer bioinformatic pipeline was used to determine bacterial community profiles. Single read sequences were trimmed and paired independently for each sample into longer fragments, after demultiplexing of the Illumina bar-coded paired reads. Non-specific amplicons were then removed and the remaining sequences were clustered through the default parameters of FROGS v1.4.0 [25] into operational taxonomic units (OTUs). Taxonomic assignment was obtained by Blast + v2.2.30 against the Silva 132 Parc database. Two steps of the swarm algorithm v2.1.6 were used to cluster the OTUs based on 97% sequence similarities. The two steps consisted of a clustering with an aggregation distance equal to 1 and of a clustering with an aggregation distance equal to 3. OTUs were removed when their relative abundance was lower than 0.005% of the whole dataset of reads. The reads are publicly available in the European Nucleotide Archive (ENA). Accession number is: PRJEB46474.

We assessed the potential bacterial DNA contamination from environment and reagents through the inclusion of various negative controls and found that the background noise and blood contamination did not impact the microbiome analysis.

### Statistical analyses

We computed the odds ratios (ORs) of CRC and IA and the corresponding 95% confidence intervals (CIs) for medium/high compared to null/low consumption of garlic using logistic regression models conditioned on study center, sex, age, and adjusted for education, energy intake, BMI, alcohol consumption and smoking habits. Further adjustments included vegetable and fruit intakes. Moreover, we computed the ORs of colon and rectal cancers separately and the ORs among strata of sex, age (< 70 and ≥ 70 years), and education (< 12 and ≥ 12 years). We also carried out sensitivity analysis by excluding outliers in energy intake. Since this yielded virtually identical results, the estimates from the whole sample are presented.

Analysis on taxonomic variables were computed among 296 subjects (because of 4 missing data in taxonomic profiling analysis) [20]. Two-tailed Mann–Whitney test was used to compare the distributions of 16S rRNA gene copies and taxa abundances between medium/high and low/null garlic consumption. Differences were also evaluated in terms of presence or absence of bacterial taxa in samples through the chi-square test. We selected taxa with a representation of at least 5% from our sample (≥ 15 subjects) and with at least a *p*-value < 0.1. We reported nominal *p* values and considered associations adjusting through the Benjamini and Hochberg method.

To assess the diversity of samples in terms of richness and evenness, Observed, Chao1, Shannon, Simpson and InvSimpson alpha-diversity indices were calculated by R PhyloSeq v1.14.0 package. Two-tailed Mann–Whitney test was used to assess their differences between the two garlic consumption groups.

## Results

Table 1 shows the distribution of healthy controls, IA and CRC patients according to selected characteristics. By design, they had the same center and sex distribution and were similar in terms of age. CRC patients tended to be less educated then the other two groups although in the absence of significant heterogeneity (*χ*^2^ test *p* = 0.155).

**Table 1:** Distribution of 100 healthy controls, 100 intestinal adenoma (IA) patients and 100 colorectal cancer (CRC) cases by study center, sex, age and years of education. Italy 2017–2019

Characteristic	Controls	IA	CRC
Study center			
Niguarda	65 (65.0%)	65 (65.0%)	65 (65.0%)
Policlinico	35 (35.0%)	35 (35.0%)	35 (35.0%)
Sex			
Male	62 (62.0%)	62 (62.0%)	62 (62.0%)
Female	38 (38.0%)	38 (38.0%)	38 (38.0%)
Age group (years)			
< 50	7 (7.0%)	4 (4.0%)	10 (10.0%)
50–59	23 (23.0%)	20 (20.0%)	19 (19.0%)
60–69	26 (26.0%)	36 (36.0%)	29 (29.0%)
≥ 70	44 (44.0%)	40 (40.0%)	42 (42.0%)
*χ*^2^ test (*p* = 0.62)			
Mean (SD) age (years)^a^	66.0 (11.8)	65.9 (10.9)	66.1 (11.6)
Education (years)^b^			
< 7	12 (12.0%)	19 (19.0%)	25 (25.0%)
7–11	24 (24.0%)	25 (25.0%)	25 (25.0%)
≥ 12	64 (64.0%)	56 (56.0%)	50 (50.0%)
*χ*^2^ test (*p* = 0.155)			

^a^Anova test for heterogeneity *p* = 1.00. SD: standard deviation (SD)

^b^The sum does not add up to the total because of one missing value

Table 2 gives the distribution of subjects, the ORs and the corresponding 95% CIs according to a medium/high versus a low/null consumption of garlic. Medium/high versus low/null garlic consumption was associated to a non-significant reduced risk of IA (OR = 0.47; 95% CI = 0.21–1.04) and to a reduced risk of CRC (OR = 0.27; 95% CI = 0.11–0.66). When CRCs and IAs were analyzed together, the OR was 0.39 (95% CI = 0.20–0.77).

**Table 2:** Odds ratios (ORs) and 95% confidence intervals (CIs) for garlic consumption in 100 colorectal cancer (CRC) cases (50 colon cancer and 50 rectal cancer cases), 100 intestinal adenomas (IA) and 100 healthy controls. Italy 2017–2019

	Garlic consumption	
	Low/null	Medium/high
Controls *n *(%)	20 (20%)	80 (80%)
IA *n *(%)	33 (33%)	67 (67%)
OR^a^	1^c^	0.43
(95% CI)		(0.21–0.91)
OR^b^	1^c^	0.47
(95% CI)		(0.21–1.04)
CRC *n *(%)^d^	34 (34.3%)	65 (65.7%)
OR^a^	1^c^	0.44
(95% CI)		(0.22–0.89)
OR^b^	1^c^	0.27
(95% CI)		(0.11–0.66)
IA and CRC *n *(%)^d^	67 (33.7%)	132 (66.3%)
OR^a^	1^c^	0.46
(95% CI)		(0.25–0.84)
OR^b^	1^c^	0.39
(95% CI)		(0.20–0.77)

^a^Estimates from logistic regression model conditioned on study center, sex and age

^b^Estimates from the model above, further adjusted for education, energy intake, BMI, alcohol consumption and smoking habit

^c^Reference

^d^Colorectal cancer cases analyzed for garlic consumption are 99 instead of 100, due to a missing value

Table 3 gives the distribution of colon and rectal cancers separately and the corresponding ORs and 95% CIs according to garlic consumption. There were 50 colon and 49 rectal cancers. The OR of colon cancer for a medium/high versus a low/null intake of garlic was 0.38 (95% CI = 0.10–1.47), whereas the corresponding OR of rectal cancer was 0.14 (95% CI = 0.03–0.61).

**Table 3:** Odds ratios (ORs) and 95% confidence intervals (CIs) for garlic consumption in 50 colon cancer cases, 50 rectal cancer cases and 100 healthy controls. Italy 2017–2019

	Garlic consumption	
	Low/null	Medium/high
Controls *n*(%)	20 (20%)	80 (80%)
Colon cancers *n* (%)	14 (28%)	36 (72%)
OR^a^	1^c^	0.67
(95% CI)		(0.23–1.87)
OR^b^	1^c^	0.38
(95% CI)		(0.10–1.47)
Rectal cancers *n*(%)^d^	20 (40.8%)	29 (59.2%)
OR^a^	1^c^	0.31
(95% CI)		(0.11–0.85)
OR^b^	1^c^	0.14
(95% CI)		(0.03–0.61)

^a^Estimates from logistic regression model conditioned on study center, sex and age

^b^Estimates from the model above, further adjusted for education, energy intake, BMI, alcohol consumption and smoking habit

^c^Reference

^d^Rectal cancer cases analyzed for garlic consumption are 49 instead of 50, due to a missing value

We computed the ORs of IA/CRC among strata of sex, age and education (data not shown). The association was similar for men and women (OR = 0.44 and OR = 0.27, respectively), whereas it appeared stronger in subjects < versus ≥ 70 years old (OR = 0.25 versus OR = 0.61, respectively) and in subjects with < 12 versus ≥ 12 years of education (OR = 0.25 versus OR = 0.58, respectively).

No significant difference was found in subjects with medium/high versus null/low garlic consumption for 16S rRNA gene copies (median of 7350 versus 6958; *p* = 0.185) nor for all the alpha-diversity indices considered, including observed (median of 29 versus 29; *p* = 0.605) and Chao1 indices (median of 43.5 versus 46.9; *p* = 0.360) at genus level (data not shown).

Tables 4 and 5 show the distribution of relative abundances and prevalence of each phylum and selected taxa, as well as *p* for tests comparing medium/high versus low/null garlic consumption. Subjects with medium/high garlic consumption had reduced relative abundance and prevalence of Patescibacteria (*p* = 0.047 and *p* = 0.049, respectively) and tended to have a higher abundance of Bacteroidetes phylum (*p* = 0.121) (Table 4).

**Table 4:** Distributions of relative abundance and prevalence of taxa at Phylum level in blood according to garlic consumption. Italy 2017–2019

Phylum	Mean		Median (I–III quartiles)		Mann–Whitney *p*-value	Prevalence (%)		${\upchi }_{1}^{2}$ *p*-value
	Low/null garlic consumption (*n* = 86)	Medium/high garlic consumption (*n* = 209)	Low/null garlic consumption (*n* = 86)	Medium/high garlic consumption (*n* = 209)		Low/null garlic consumption (*n* = 86)	Medium/high garlic consumption (*n* = 209)	
Actinobacteria	18.3	17.3	16.5 (11.6–23.4)	15.5 (11.1–22.9)	0.434	86 (100)	209 (100)	-
Bacteroidetes	7.1	8.2	4.5 (1.5–9.9)	6.7 (3.6–11.6)	0.121	85 (98.8)	204 (97.6)	0.497
Chlamydiae	0.1	0.3	0.0 (0.0–0.0)	0.0 (0.0–0.0)	0.392	25 (29.1)	69 (33.0)	0.509
Dependentiae	0.2	0.2	0.0 (0.0–0.0)	0.0 (0.0–0.0)	0.622	11 (12.8)	31 (14.8)	0.648
Firmicutes	8.1	6.7	5.4 (0.0–11.9)	4.8 (0.0–10.4)	0.762	82 (95.3)	201 (96.2)	0.745
Patescibacteria	0.5	0.3	0.0 (0.0–0.0)^a^	0.0 (0.0–0.0)	0.047*	24 (27.9)^b^	37 (17.7)	0.049*
Proteobacteria	65.4	66.6	66.9 (59.2–75.1)	67.9 (59.8–74.8)	0.884	86 (100)	209 (100)	-

*p < 0.05

^a^Higher abundance where *p* < 0.05

^b^Higher prevalence where *p* < 0.05

**Table 5:** Distributions of relative abundance and prevalence of selected taxa in blood according to garlic consumption. Italy 2017–2019

Taxa (Phylum; class; order; family; genus)	Mean		Median (I–III quartiles)		Mann–Whitney *p*-value	Prevalence (%)		${\upchi }_{1}^{2}$ *p*-value
	Low/null garlic consumption (*n* = 86)	Medium/high garlic consumption (*n* = 209)	Low/null garlic consumption (*n* = 86)	Medium/high garlic consumption (*n* = 209)		Low/null garlic consumption (*n* = 86)	Medium/high garlic consumption (*n* = 209)	
Actinobacteria; Actinomycetia; Corynebacteriales (order)	3.5	3.7	2.0 (0.0–5.8)	2.5 (0.0–5.5)	0.526	70 (81.4)	188 (90.0)^b^	0.044*
Actinobacteria; Actinomycetia; Corynebacteriales; Nocardiaceae (family)	1.5	1.4	0.0 (0.0–1.7)	0.0 (0.0–1.6)	0.158	45 (52.3)	141 (67.5)^b^	0.014*
Actinobacteria; Actinomycetia; Corynebacteriales; Nocardiaceae; Rhodococcus (genus)	1.5	1.3	0.0 (0.0–1.7)	0.0 (0.0–1.0)	0.291	45 (52.3)	136 (65.1)^b^	0.041*
Actinobacteria; Actinomycetia; Frankiales; Geodermatophilaceae; Modestobacter (genus)	1.0	0.0	0.0 (0.0–0.0)^a^	0.0 (0.0–0.0)	0.003*	13 (15.1)^b^	11 (5.3)	0.005*
Actinobacteria; Actinomycetia; Micrococcales; Micrococcaceae (family)	5.7	7.2	5.3 (2.9–8.4)	6.4 (3.5–9.7)	0.051	85 (98.8)	207 (99.0)	0.873
Actinobacteria; Actinomycetia; Micrococcales; Micrococcaceae; Rothia (genus)	0.1	0.6	0.0 (0.0–0.0)	0.0 (0.0–0.0)^a^	0.024*	6 (7.0)	35 (16.7)^b^	0.027*
Firmicutes; Bacilli; Bacillales; Bacillaceae (family)	1.2	0.6	0.0 (0.0–0.0)^a^	0.0 (0.0–0.0)	0.017*	35 (40.7)^b^	53 (25.4)	0.009*
Firmicutes; Bacilli; Bacillales; Bacillaceae; Bacillus (genus)	0.8	0.4	0.0 (0.0–0.0)	0.0 (0.0–0.0)	0.054	25 (29.1)^b^	38 (18.2)	0.038*
Firmicutes; Clostridia; Clostridiales; Family XI (family)	0.6	0.3	0.0 (0.0–0.0)^a^	0.0 (0.0–0.0)	0.009*	24 (27.9)^b^	30 (14.4)	0.006*
Firmicutes; Clostridia; Clostridiales; Family XI; Anaerococcus (genus)	0.3	0.1	0.0 (0.0–0.0)	0.0 (0.0–0.0)	0.068	9 (10.5)	10 (4.8)	0.071
Firmicutes; Clostridia; Clostridiales; Family XI; Finegoldia (genus)	0.3	0.0	0.0 (0.0–0.0)^a^	0.0 (0.0–0.0)	< 0.001*	13 (15.1)^b^	7 (3.3)	< 0.001*
Patescibacteria; Saccharimonadia (class)	0.4	0.2	0.0 (0.0–0.0)^a^	0.0 (0.0–0.0)	0.021*	18 (20.9)^b^	22 (10.5)	0.018*
Patescibacteria; Saccharimonadia; Saccharimonadales (order)	0.4	0.2	0.0 (0.0–0.0)^a^	0.0 (0.0–0.0)	0.021*	18 (20.9)^b^	22 (10.5)	0.018*
Proteobacteria; Alphaproteobacteria; Caulobacterales (order)	5.0	4.1	3.4 (0.1–7.0)	2.8 (0.0–6.2)	0.096	85 (98.8)^b^	193 (92.3)	0.030*
Proteobacteria; Alphaproteobacteria; Caulobacterales; Caulobacteraceae (family)	5.0	4.0	3.4 (0.1–7.0)	2.7 (0.0–6.2)	0.079	85 (98.8)^b^	193 (92.3)	0.030*
Proteobacteria; Alphaproteobacteria; Caulobacterales; Caulobacteraceae; Brevundimonas (genus)	0.1	0.3	0.0 (0.0–0.0)	0.0 (0.0–0.0)	0.060	9 (10.5)	41 (19.6)	0.057
Proteobacteria; Alphaproteobacteria; Rickettsiales; SM2D12 (family)	0.3	0.5	0.0 (0.0–0.0)	0.0 (0.0–0.0)	0.054	13 (15.1)	53 (25.4)	0.055
Proteobacteria; Gammaproteobacteria; Betaproteobacteriales; Burkholderiaceae (family)	7.5	6.4	6.0 (3.4–9.3)^a^	4.6 (2.4–8.4)	0.038*	86 (100)	206 (98.6)	0.264
Proteobacteria; Gammaproteobacteria; Betaproteobacteriales; Burkholderiaceae; Cupriavidus (genus)	0.4	0.6	0.0 (0.0–0.0)	0.0 (0.0–0.0)	0.084	17 (19.8)	60 (28.7)	0.112
Proteobacteria; Gammaproteobacteria; Betaproteobacteriales; Burkholderiaceae; Polynucleobacter (genus)	0.2	0.1	0.0 (0.0–0.0)^a^	0.0 (0.0–0.0)	0.039*	12 (14.0)^b^	14 (6.7)	0.046*
Proteobacteria; Gammaproteobacteria; Betaproteobacteriales; Neisseriaceae (family)	0.3	0.4	0.0 (0.0–0.0)	0.0 (0.0–0.0)	0.104	12 (14.0)	47 (22.5)	0.096
Proteobacteria; Gammaproteobacteria; Oceanospirillales (order)	0.4	0.1	0.0 (0.0–0.0)^a^	0.0 (0.0–0.0)	0.042*	12 (14.0)^b^	14 (6.7)	0.046*
Proteobacteria; Gammaproteobacteria; Oceanospirillales; Halomonadaceae (family)	0.3	0.1	0.0 (0.0–0.0)	0.0 (0.0–0.0)	0.084	11 (12.8)	14 (6.7)	0.088
Proteobacteria; Gammaproteobacteria; Oceanospirillales; Halomonadaceae; Halomonas (genus)	0.3	0.1	0.0 (0.0–0.0)	0.0 (0.0–0.0)	0.084	11 (12.8)	14 (6.7)	0.088
Proteobacteria; Deltaproteobacteria; Bdellovibrionales (order)	0.3	0.2	0.0 (0.0–0.0)	0.0 (0.0–0.0)	0.083	10 (11.6)	12 (5.7)	0.080
Proteobacteria; Deltaproteobacteria; Bdellovibrionales; Bdellovibrionaceae (family)	0.3	0.1	0.0 (0.0–0.0)	0.0 (0.0–0.0)	0.061	8 (9.3)	8 (3.8)	0.059
Proteobacteria; Deltaproteobacteria; Bdellovibrionales; Bdellovibrionaceae; Bdellovibrio (genus)	0.3	0.1	0.0 (0.0–0.0)^a^	0.0 (0.0–0.0)	0.034*	8 (9.3)^b^	7 (3.3)	0.034*

*p < 0.05

^a^Higher abundance where *p* < 0.05

^b^Higher prevalence where *p* < 0.05

According to phylogenetic branches, prevalence was higher in the medium/high garlic consumption group for Corynebacteriales order (*p* = 0.044), Nocardiaceae family (*p* = 0.014) and *Rhodococcus* genus (*p* = 0.041). Relative abundances were higher in the medium/high garlic consumption group for *Rothia* genus (*p* = 0.024) and lower for *Modestobacter* genus (*p* = 0.003).

Among Firmicutes, relative abundances were lower in the medium/high garlic consumption for Bacillaceae family (*p* = 0.017), Family XI (*p* = 0.009) and *Finegoldia* genus (p < 0.001). Also prevalence was lower for these taxa (*p* = 0.009, *p* = 0.006 and *p* < 0.001, respectively) and for *Bacillus* genus (*p* = 0.038).

Prevalence was lower in the medium/high garlic consumption group for Caulobacterales order (*p* = 0.030) and Caulobacteraceae family (*p* = 0.030).

Relative abundances were lower for Burkholderiaceae family (*p* = 0.038) and *Polynucleobacter* genus (*p* = 0.039).

Relative abundances were also lower for Oceanospirillales order (p = 0.042), among Gammaproteobacteria class, and *Bdellovibrio* genus (*P* = 0.034), among Deltaproteobactria class.

Results on taxa belonging to Corynobacteriales and Clostridiales (Family XI) orders were confirmed also after restricting the analyses to controls only (Supplementary Table 1).

## Discussion

The present study indicates that the consumption of garlic reduced the risk of CRC and IA and was apparently associated with selected features of bacterial DNA in blood. In particular, garlic was directly related to the carriage of selected taxa belonging to Corynebacteriales and inversely related to relative abundances of taxa belonging to Clostridiales.

Our results on garlic consumption and CRC and IA agree with a meta-analysis [7] on 7 studies (10,026 cases) evaluating garlic consumption and CRC risk, which found a pooled RR of 0.85 (95% CI = 0.72–1.00) for high garlic consumption. That meta-analysis found that the intake of total allium vegetables (among which garlic) was also inversely related to IA risk, with a pooled RR of 0.88 (95% CI = 0.80–0.98), based on three studies (4,873 cases). After that meta-analysis, another study conducted in China investigated garlic consumption in relation to CRC risk among 833 cases and as many controls matched by age, sex and residence area [26]. The study reported an OR for the highest versus the lowest category of garlic intake (> 3.65 versus < 0.60 kg/years) of 0.56 (95% CI = 0.39–0.79). Our results are in line with those data, although they appear stronger. This could be due to the traditional use of garlic with other healthy products as extra virgin olive oil, tomato and other vegetables in the Italian diet [27]. Our results, which suggest a protective effect of garlic on CRC, however, were confirmed also after adjusting for possible healthy diet confounders, namely vegetable and fruit intake (Supplementary Table 2).

According to CRC subsites the meta-analysis reported a RR of 0.90 (95% CI = 0.75–1.08) for colon cancer and of 0.76 (95% CI = 0.59–0.98) for rectal cancer, while the Chinese study reported an OR of 0.49 (95% CI = 0.27–0.90) for proximal colon cancer, 0.57 (95% CI = 0.32–1.01) for distal colon cancer and 0.47 (95% CI = 0.31–0.71) for rectal cancer. These findings are also in line with our results that found a stronger risk reduction for rectal than colon cancer.

Stratification by sex showed no substantial differences between men and women in the meta-analysis nor in our data. Results by age and education were not available in the meta-analysis, whereas our data showed a stronger CRC risk reduction for higher garlic consumption in < 70 years old and in less educated subjects. However, our results on strata should be interpreted with caution given the small number of subjects.

Garlic effect on CRC risk reduction can be explained by its content in fiber (inulin-type fructans in particular), polyphenols and organosulfur compounds [9], that can also influence recognized pathogenetic factors for CRC such as immunity, inflammation, metabolic conditions and microbiota [28–30]. Fructans are soluble fibers with prebiotic activity [31]. They also exert immunomodulatory activity, as their fermentation by the colonic microbiota results in short chain fatty acids (SCFAs). A study on murine CD4^+^ T cells described that microbiota derived SCFAs are able to increase intestinal IL-22 production, which protects the intestine from inflammation and acts as a regulator for intestinal homeostasis and barrier function [32]. Another study on patients with ulcerative colitis found inulin-type fructans to be possibly beneficial in reducing symptoms [33]. A randomized trial showed that a 20 g/day supplementation of inulin improved insulin sensibility compared to cellulose, indicating a possible influence of inulin on metabolic pathways [34]. In the latter study, inulin supplementation was also associated with gut bacterial changes. In particular, inulin supplementation was associated to an increase in Actinobacteria and to a decrease in Clostridia at class level, and a decrease in Clostridiales at order level. Another trial found that inulin-type-fructans were associated to a decreased level of Clostridiales [35]. Those results are consistent with our findings in which higher garlic consumption was associated with increased prevalence of Corynebacteriales order, Nocardiaceae family and *Rhodococcus* genus, belonging to Actinomycetia (formerly Actinobacteria) class, and with reduced abundance and prevalence of Family XI and *Finegoldia* genus, belonging to the Clostridia class and Clostridiales order. Referring to polyphenols, a RCT showed that polyphenol-rich dietary pattern reduced the serum levels of zonulin, which is considered a marker of intestinal permeability [36]. Moreover, quercetin is known for its anti-inflammatory, anticarcinogenic and anti-diabetic effect [37, 38] and was able to reduce plasmatic methylglyoxal, a precursor of advanced glycation end products, in a RCT on 37 subjects [39]. Oil-soluble organosulfur compounds in garlic include allicin, ajoenes, and allyl sulfides and showed a range of antibacterial properties such as bactericidal, antibiofilm and antitoxin [40]. Moreover, a RCT on 49 subjects with hypertension found that aged-garlic-extract supplementation influenced stool microbiota when compared to placebo, by increasing microbial richness and diversity and by producing a shift of bacterial species of the Firmicutes phylum [41].

Inflammation, insulin resistance, metabolic syndrome and type 2 diabetes have been related to CRC [42–44] and other conditions associated to changes in human microbiota [45–51]. Corynebacteriales order was more prevalent in the medium/high versus low/null garlic consumption in our data, and in a recent prospective study using 16S rRNA analysis for blood microbiome assessment, Corynebacteriales were found to be inversely related with inflammatory cytokines among 32 patients with portal hypertension [48]. Corynobacteriales order, Nocardiaceae family and *Rhodococcus* genus in fecal microbiota were also inversely associated to gestational diabetes risk in a study on pregnant women [49]. According to our data, lower abundance of Family XI has been found in stool samples in healthy as compared to polycystic ovary syndrome adolescent girls [50]. In that study, a reduction of Family XI was also associated to lower hepato-visceral fat and to treatment with spironolactone-pioglitazone-metformin. In a study on skin microbiome, *Finegoldia* abundance was found lower and Corynebacteriales abundance was found higher in healthy volunteers than in patients with psoriasis [51].

To sum up, the differences that we found in bacterial taxa in blood according to garlic consumption were similar to those reported in literature for pathologic conditions associated to insulin resistance and/or glucose intolerance [45–47]. This, together with the fact that garlic and some of its components show anti-diabetic and metabolic ameliorating properties [14, 52–54], may suggest a role of these taxa as indicators for insulin resistance and/or glucose intolerance.

One of the limits of our study is the absence of other microbiota samples (e.g., skin, oral, fecal microbiota), which did not allow us to analyze thoroughly the mechanisms related to the effect of garlic on bacterial translocation and CRC development. Size or shape selectivity for bacteria transiting from different locations to bloodstream could influence our results. When considering multiple test adjustment, differences in terms of microbiota data according to garlic consumption groups lost their significance, but the low sample size remains a major limitation of our study. However, our results are broadly in line with previous findings involving different microbiota locations and pathological conditions [48–51]. Other limitations are related to the case–control study design [55]. With relation to selection bias, we conducted an ad-hoc data collection and developed a protocol including standardized procedures to interview all patients admitted to target hospitals for colonoscopy. Moreover, cases and controls were matched by study center, sex and age, and were interviewed in similar settings. Our cases were truly incident as they were detected at the first CRC-diagnosing colonoscopy. Thus, they had minimal time between recruitment and diagnosis, avoiding possible habit modifications in the recent past, and had available clinical data throughout the whole diagnostic process. Moreover, healthy controls allowed a clean comparison with CRC and IA, and the inclusion of IA granted the possibility to deeper explore the adenoma-carcinoma sequence.

Interviewers and investigators were blinded to information on CRC, IA and healthy group assignment, also during the metataxonimic analyses. We avoided possible contamination during colonoscopy, by collecting blood samples before the procedure. We took care to keep optimal signal to noise ratio and reduce technical variability, by analyzing all blood samples in the same experiment with the same reagent batches and manipulator.

Our questionnaire was administered by trained interviewers, to reduce problems of incorrect compiling and misinterpretation. Interviews were performed before colonoscopy (and group assignment), thus avoiding any influence from cancer diagnosis. Data from our FFQ were satisfactorily reproducible and valid [23]. For frequency of garlic use, we asked for the common consumption as an ordinal qualitative variable, and no additional information on manner of using and on the form of dietary garlic (e.g., fresh, cooked or powder) was available. The latter aspect can be a limitation since anticancer and antioxidant activities can vary with respect to different garlic forms and preparations [56].

With reference to confounding, we were able to adjust for study center, sex and age, and for other confounders including energy intake, BMI, smoking and alcohol consumption, as well as for vegetable and fruit intake.

This research highlights the importance of diet and the potential role of garlic in CRC prevention. Moreover, this is, to our knowledge, the first study to investigate the relation between dietary garlic use and bacterial DNA in blood, corroborating the hypothesis of diet-microbiota axis as a mechanism behind this relation. The study provides supporting evidence to the existence of translocation of bacterial material from some body areas to bloodstream. The differences we found in bacterial taxa prevalence and abundance according to garlic consumption suggest further investigations on the existence of possible blood bacterial DNA markers for unhealthy diets or diseases, possibly aiming to personalize treatment.

## Supplementary Information

Below is the link to the electronic supplementary material.

Supplementary file1 (DOCX 25 KB)

## Data Availability

All the reads are publicly available in the European Nucleotide Archive (ENA) with the accession number: PRJEB46474.
