# Supplementary material for: Garlic consumption in relation to colorectal cancer risk and to alterations of blood bacterial DNA
Source: Eur J Nutr. 2023 Apr 24;62(5):2279–92. doi: 10.1007/s00394-023-03110-2 (PMC10349700; doi:10.1007/s00394-023-03110-2)
Supplement: Supplementary file 1 — Supplementary file1 (DOCX 25 KB) [file 394_2023_3110_MOESM1_ESM.docx]

**Supplementary table 1** Distributions of relative abundance and prevalence of selected taxa in blood according to garlic consumption among controls. Italy 2017-2019.

| **Taxa (Phylum; class; order; family; genus)** | **Mean** | | **Median (I-III quartiles)** | | **Mann Whitney**  **p-value** | **Prevalence of taxa (%)** | | $\boldsymbol{\chi}_{\mathbf{1}}^{\mathbf{2}}$  **p-value** |
| --- | --- | --- | --- | --- | --- | --- | --- | --- |
|  | **low/null**  **garlic consumption**  **(n=19)** | **medium/high**  **garlic consumption**  **(n=79)** | **low/null**  **garlic consumption**  **(n=19)** | **medium/high**  **garlic consumption**  **(n=79)** |  | **low/null**  **garlic consumption**  **(n=19)** | **medium/high**  **garlic consumption**  **(n=79)** |  |
| Actinobacteria; Actinomycetia; Corynebacteriales (order) | 4,1 | 3,4 | 2,6 (0,0-8,4) | 2,8 (0,0-4,9) | 0,770 | 14 (73.7) | 72 (91.1)^b^ | 0,037* |
| Actinobacteria; Actinomycetia; Corynebacteriales; Nocardiaceae (family) | 1,8 | 1,3 | 0,0 (0,0-2,5) | 0.0 (0.0-0.7) | 0,280 | 8 (42,1) | 53 (67.1)^b^ | 0,044* |
| Actinobacteria; Actinomycetia; Corynebacteriales; Nocardiaceae; Rhodococcus (genus) | 1,8 | 1,2 | 0,0 (0,0-2,5) | 0.0 (0.0-0.3) | 0,347 | 8 (42,1) | 52 (65.8) | 0,057 |
| Actinobacteria; Actinomycetia; Frankiales; Geodermatophilaceae; Modestobacter (genus) | 0,0 | 0,0 | 0.0 (0.0-0.0) | 0.0 (0.0-0.0) | 0,944 | 1 (5.3) | 4 (5.1) | 0,972 |
| Actinobacteria; Actinomycetia; Micrococcales; Micrococcaceae (family) | 5,2 | 7,3 | 4,2 (2,8-6,2) | 6,6 (3,5-10,1) | 0,081 | 19 (100) | 79 (100) | - |
| Actinobacteria; Actinomycetia; Micrococcales; Micrococcaceae; Rothia (genus) | 0,0 | 0,4 | 0.0 (0.0-0.0) | 0.0 (0.0-0.0) | 0,215 | 1 (5.3) | 13 (16.5) | 0,211 |
| Firmicutes; Bacilli; Bacillales; Bacillaceae (family) | 0,7 | 0,5 | 0.0 (0.0-0.0) | 0.0 (0.0-0.0) | 0,649 | 5 (26.3) | 17 (21.5) | 0,653 |
| Firmicutes; Bacilli; Bacillales; Bacillaceae; Bacillus (genus) | 0,7 | 0,5 | 0.0 (0.0-0.0) | 0.0 (0.0-0.0) | 0,373 | 5 (26.3) | 13 (16.5) | 0,319 |
| Firmicutes; Clostridia; Clostridiales; Family XI (family) | 1,5 | 0,2 | 0.0 (0.0-1.8)^a^ | 0.0 (0.0-0.0) | 0,032* | 7 (36.8)^b^ | 13 (16.5) | 0,048* |
| Firmicutes; Clostridia; Clostridiales; Family XI; Anaerococcus (genus) | 0,4 | 0,0 | 0.0 (0.0-0.0) | 0.0 (0.0-0.0) | 0,778 | 1 (5.3) | 6 (7.6) | 0,723 |
| Firmicutes; Clostridia; Clostridiales; Family XI; Finegoldia (genus) | 0,9 | 0,0 | 0.0 (0.0-0.0)^a^ | 0.0 (0.0-0.0) | 0,001* | 5 (26.3)^b^ | 3 (3.8) | 0,001* |
| Patescibacteria; Saccharimonadia (class) | 0,5 | 0,3 | 0.0 (0.0-0.0) | 0.0 (0.0-0.0) | 0,592 | 3 (15.8) | 9 (11.4) | 0,600 |
| Patescibacteria; Saccharimonadia; Saccharimonadales (order) | 0,5 | 0,3 | 0.0 (0.0-0.0) | 0.0 (0.0-0.0) | 0,592 | 3 (15.8) | 9 (11.4) | 0,600 |
| Proteobacteria; Alphaproteobacteria; Caulobacterales (order) | 5,2 | 4,2 | 4,5 (1,1-7,0) | 2,3 (0,0-6,0) | 0,196 | 19 (100) | 71 (89.9) | 0,148 |
| Proteobacteria; Alphaproteobacteria; Caulobacterales; Caulobacteraceae (family) | 5,2 | 4,1 | 4,5 (1,1-7,0) | 1,9 (0,0-6,0) | 0,172 | 19 (100) | 71 (89.9) | 0,148 |
| Proteobacteria; Alphaproteobacteria; Caulobacterales; Caulobacteraceae; Brevundimonas (genus) | 0,2 | 0,3 | 0.0 (0.0-0.0) | 0.0 (0.0-0.0) | 0,523 | 3 (15.8) | 18 (22.8) | 0,505 |
| Proteobacteria; Alphaproteobacteria; Rickettsiales; SM2D12 (family) | 0,0 | 0,5 | 0.0 (0.0-0.0) | 0.0 (0.0-0.0) | 0,100 | 2 (10.5) | 21 (26.6) | 0,138 |
| Proteobacteria; Gammaproteobacteria; Betaproteobacteriales; Burkholderiaceae (family) | 8,8 | 8,3 | 7,7 (3,2-12,3) | 6,1 (3,5-10,2) | 0,653 | 19 (100) | 79 (100) | - |
| Proteobacteria; Gammaproteobacteria; Betaproteobacteriales; Burkholderiaceae; Cupriavidus (genus) | 1,0 | 0,9 | 0.0 (0.0-0.0) | 0.0 (0.0-0.0) | 0,199 | 3 (15.8) | 25 (31.6) | 0,170 |
| Proteobacteria; Gammaproteobacteria; Betaproteobacteriales; Burkholderiaceae; Polynucleobacter (genus) | 0,1 | 0,1 | 0.0 (0.0-0.0) | 0.0 (0.0-0.0) | 0,381 | 2 (10.5) | 4 (5.1) | 0,373 |
| Proteobacteria; Gammaproteobacteria; Betaproteobacteriales; Neisseriaceae (family) | 0,0 | 0,2 | 0.0 (0.0-0.0) | 0.0 (0.0-0.0) | 0,124 | 1 (5.3) | 16 (20.3) | 0,121 |
| Proteobacteria; Gammaproteobacteria; Oceanospirillales (order) | 0,1 | 0,2 | 0.0 (0.0-0.0)^a^ | 0.0 (0.0-0.0) | 0,046* | 4 (21.1)^b^ | 5 (6.3) | 0,046* |
| Proteobacteria; Gammaproteobacteria; Oceanospirillales; Halomonadaceae (family) | 0,1 | 0,2 | 0.0 (0.0-0.0)^a^ | 0.0 (0.0-0.0) | 0,046* | 4 (21.1)^b^ | 5 (6.3) | 0,046* |
| Proteobacteria; Gammaproteobacteria; Oceanospirillales; Halomonadaceae; Halomonas (genus) | 0,1 | 0,2 | 0.0 (0.0-0.0)^a^ | 0.0 (0.0-0.0) | 0,046* | 4 (21.1)^b^ | 5 (6.3) | 0,046* |
| Proteobacteria; Deltaproteobacteria; Bdellovibrionales (order) | 0,2 | 0,1 | 0.0 (0.0-0.0) | 0.0 (0.0-0.0) | 0,248 | 2 (10.5) | 3 (3.8) | 0,231 |
| Proteobacteria; Deltaproteobacteria; Bdellovibrionales; Bdellovibrionaceae (family) | 0,2 | 0,0 | 0.0 (0.0-0.0) | 0.0 (0.0-0.0) | 0,119 | 2 (10.5) | 2 (2.5) | 0,114 |
| Proteobacteria; Deltaproteobacteria; Bdellovibrionales; Bdellovibrionaceae; Bdellovibrio (genus) | 0,2 | 0,0 | 0.0 (0.0-0.0) | 0.0 (0.0-0.0) | 0,119 | 2 (10.5) | 2 (2.5) | 0,114 |

^*^ p<0.05.

^a^ Higher abundance where p<0.05.

^b^ Higher prevalence where p<0.05.

**Supplementary table 2** Odds ratios (ORs) and 95% confidence intervals (CIs) for garlic consumption in 100 colorectal cancer (CRC) cases or/and 100 intestinal adenomas (IA) compared to100 healthy controls. Italy 2017-2019.

|  | **garlic consumption** | |
| --- | --- | --- |
|  | **low/null** | **medium/high** |
| **IA** |  |  |
| OR^a^ | 1^b^ | 0.45 |
| (95% CI) |  | (0.20-1.02) |
| OR^c^ | 1^b^ | 0.41 |
| (95% CI) |  | (0.18-0.94) |
| OR^d^ | 1^b^ | 0.46 |
| (95% CI) |  | (0.20-1.06) |
| **CRC^e^** |  |  |
| OR^a^ | 1^b^ | 0.24 |
| (95% CI) |  | (0.10-0.62) |
| OR^c^ | 1^b^ | 0.27 |
| (95% CI) |  | (0.11-0.66) |
| OR^d^ | 1^b^ | 0.21 |
| (95% CI) |  | (0.08-0.57) |
| **IA and CRC^e^** |  |  |
| OR^a^ | 1^b^ | 0.33 |
| (95% CI) |  | (0.18-0.71) |
| OR^c^ | 1^b^ | 0.37 |
| (95% CI) |  | (0.19-0.74) |
| OR^d^ | 1^b^ | 0.35 |
| (95% CI) |  | (0.18-0.71) |

^a^ Estimates from the model conditioned on study center, sex and age, and further adjusted for education, energy intake, BMI, alcohol consumption, smoking habit, and fruit consumption.

^b^ Reference.

^c^ Estimates from the model conditioned on study center, sex and age, and further adjusted for education, energy intake, BMI, alcohol consumption, smoking habit, and vegetables consumption.

^d^ Estimates from the model conditioned on study center, sex and age, and further adjusted for education, energy intake, BMI, alcohol consumption, smoking habit, and fruit and vegetables consumption.

^e^ Colorectal cancer cases analyzed were 99 due to a missing value.
